# An epidemiological study of respiratory syncytial virus associated hospitalizations in Denmark

**DOI:** 10.1186/rr189

**Published:** 2002-06-24

**Authors:** Lone Graff Stensballe

**Affiliations:** 1 Department of Epidemiology Research, Danish Epidemiology Science Centre, Statens Serum Institut, Copenhagen, Denmark

**Keywords:** cohort study, Denmark, epidemiology, hospitalization, respiratory syncytial virus

## Abstract

Respiratory syncytial virus (RSV) is the most common viral pathogen that causes lower respiratory tract infections in infants. Studies have implicated severe RSV infections early in life as a risk factor for subsequent development of reactive airway disease. We are conducting a study to validate RSV-associated diagnoses in the Danish National Patient Registry, to assess whether the incidence of severe RSV infection is increasing in Denmark, to identify predisposing and protective factors for RSV-associated hospitalization in Denmark, and to examine the association of severe RSV infection with reactive airway disease. The influence of various biological, social and environmental factors on hospitalization for RSV infection will be studied through several population-based registers, including the Danish National Birth Cohort: 'Better health for mothers and children'. The RSV hospitalization cases will be compared with control individuals selected within the same population groups on a case–control or a cohort basis in order to produce estimates of age-adjusted and sex-adjusted relative risks (odds ratio and relative risk) for hospitalization associated with various risk factors. Using register linkage and unique registration of exposures collected through interviews and blood samples from the Danish National Birth Cohort, we will be able to resolve the issues referred to above in a very large sample of Danish children.

## Introduction

Respiratory syncytial virus (RSV) was first isolated from American children with pulmonary disease in 1957 [1], and it is now recognized as the most important cause of viral lower respiratory tract disease in infants and children worldwide [2-4]. RSV is the largest single cause of childhood hospitalization and is therefore a major drain on public health resources [5]. Furthermore, recent data from the USA [6] suggest that rates of hospitalization due to RSV infections are increasing. A Danish study [7] found the incidence of RSV infection requiring hospitalization during the 1995–1996 winter season to be 34/1000 in infants younger than 6 months. Not many Danish RSV epidemiological studies have been conducted, however, and the few studies reported thus far were conducted in limited geographical areas [8].

High-risk groups for severe RSV infection include infants born prematurely [9]; children with congenital hearth disease [10], cystic fibrosis [11], or other chronic lung diseases [12]; immunosuppressed patients or patients with congenital immunodeficiency [13]; individuals living in institutions; and the elderly [14,15]. Hence, children discharged from neonatal intensive care units are at risk for rehospitalization from RSV infection [16]. However, the majority of infants who develop severe RSV disease were born at term and are otherwise healthy. Among those, certain factors have been associated with severe disease: month of birth, age younger than 6 months, male sex, ethnicity [17], low socioeconomic status, crowded living conditions, number of siblings, indoor smoke pollution, day-care attendance [18], and a family history of asthma and atopy [19,20]. Many studies suggest that passively acquired maternal antibody confers protection against severe RSV disease [21,22], and incomplete transfer of maternally derived antibodies has been proposed as a contributing factor to the increased risk for RSV infection that is observed in preterm infants [9]. Breast-feeding also protects against severe RSV infection [21,23].

Within the first year following severe, hospital-requiring RSV infection, up to 20% of children are rehospitalized because of wheezing [24]. RSV bronchiolitis has been associated with abnormal pulmonary function, wheezing and asthmatic tendencies in children up to age 11 years [25,26]. Hospitalization-requiring RSV bronchiolitis during the first year of life has been found to be an important risk factor in children up to age 7 years for development not only of asthma but also of sensitization to common allergens, particularly in individuals with a genetic predisposition to atopy [27].

Recent studies suggest that severe RSV infection is associated with a Th2 cell response [28] and that enhanced lung pathology during RSV infection is associated with local release of Th2 cytokines [29]. Therefore, it is likely that a Th2 profile facilitates infection and immunopathogenesis, or limits protective responses. The immune system of the newborn child is usually Th2 biased [30,31], and this may partly explain why RSV infection is particularly severe during the first 6 months of life. Likewise, atopy is associated with a Th2 profile [32,33], and this may explain why severe RSV infection is associated with a family history of atopy and asthma [19].

In recent decades there has been an increase in atopic disease, which appears to be related to an increasing Th2 bias in the immunological profile. In contrast, we found that a strong reaction (scar) to bacille Calmette-Guérin vaccination (a promoter of a Th1 profile [34]) was protective against RSV disease in Guinea-Bissau, because not having a scar was twice as common among children with RSV lower respiratory tract infection than among control children (Stensballe LG, unpublished data). Furthermore, bacille Calmette–Guérin vaccination prevents atopy [35].

A relationship between prior RSV infection, wheezing and atopic disease has frequently been reported, but it is not known whether this is a causal association or due to common determinants of the two conditions. Above all, the way in which we influence our disease patterns and how we interact with our surroundings may have consequences for our immunological profile and RSV morbidity. Recently, we found that caesarean section increases the risk for atopic disease [36], and another study [37] showed an association between early childhood use of antibiotics and increased risk for developing asthma and allergic disease in predisposed children. If the association between severe RSV infection and atopy is in part explained by a common immunological bias, then the same factors (i.e. vaccination history, caesarean section and use of antibiotics) might influence the risk for severe RSV infection.

Exposure intensity has been found to be a determinant of infection or severity of several diseases, including measles [38], chickenpox, polio and whooping cough. Crowding, large family size, multiple birth, small living quarters and day-care attendance facilitate intensive exposure, and these factors are usually found to be risk factors for severe disease. It appears likely that this also applies to RSV.

Because cross-sex transmission increases severity of infection in measles [39], chickenpox and polio [40], part of the reason why boys are more likely to contract severe infection may be that mothers are more likely to transmit RSV to their sons. In Guinea-Bissau, among children with lower respiratory tract infection, we found that boys were twice as likely as girls to have an RSV antigen positive infection if the mother had an RSV-IgM or RSV-IgA response, indicating recent infection (Stensballe LG, unpublished data).

Attempts to develop a safe and effective RSV vaccine have met with failure. Likewise, there is no treatment available against severe RSV infection. Prophylaxis with the humanized monoclonal antibody palivizumab can reduce risk for hospitalization for RSV disease in high-risk infants [41-43] , but the drug is expensive. A recent study [44] showed that this antibody can prevent RSV-induced neurogenic inflammation of mouse airways when given before or in the early phase of infection. If this also applies to humans then passive prophylaxis or immunization of pregnant women might prevent not only severe RSV infection but also wheezing.

## Research questions

### Aim 1

Our first objective is to conduct a study to validate RSV associated diagnoses in the Danish National Patient Registry (DNPR). This validation will enable us to assess whether the incidence of severe RSV is increasing. The analyses will determine the reliability of the RSV-specific categories in the DNPR and how well these cover the full range of RSV infections that lead to hospitalization. The worst case scenario would be that the DNPR data are impossible to use, and we would then have to base our epidemiological studies exclusively on patients with a positive laboratory diagnosis of RSV. It is more likely that certain clinical RSV categories are reasonably reliable but do not cover the full range of RSV infections. The study will at least provide a good basis for standardizing classification of RSV infections in the future.

### Aim 2

A prerequisite for rational use of currently available prophylactic and preventive measures and vaccines, when they become available, is a better understanding of the factors that promote or prevent severe RSV infection. Apart from examining the role of conditions that are known to be risk factors for severe RSV infections in other Western countries, we wish to examine some new factors associated with RSV infections. We will examine whether factors that facilitate transmission of viral respiratory infections, and whether caesarean section, use of antibiotics and vaccination history contribute to RSV infection. We will also measure the incidence of nosocomial RSV infection and determine risk factors for nosocomial infection. In addition, we intend to determine the level of maternal RSV antibodies in cord blood that is needed to confer protection against disease during the first critical months of life. This information will be applied to maternal vaccination programmes.

### Aim 3

We intend to examine several issues in order to address the link between severe RSV infection, wheezing and atopy: the chronological relation between RSV infections and wheezing; the incidence of rehospitalization for wheezing after RSV infection and risk factors for rehospitalization; the chronological relation between RSV infections and atopic dermatitis; whether risk factors for atopy are also risk factors for severe RSV infection; whether an atopic profile (determined as specific IgE in serum) is more common among mothers of children who are hospitalized with RSV than among mothers of control children; whether the level of total IgE in cord blood is an indicator of atopic profile among children hospitalized with RSV in comparison with control children; and whether a high level of maternal RSV antibodies in cord blood is protective against RSV hospitalization and wheezing.

## Materials and method

### The personal identification number

Since 1968 a personal identification number has been assigned to every Danish citizen and registered in the Central Person Register (CPR). This personal identification number provides a unique ability for epidemiological research in the Danish population by facilitating reliable linkage of person-identifiable information between various registers. The personal identification number serves as the key reference to the individual in all registry relations and provides a simple method for linking registry information.

### Validation of the diagnostic coding in the Danish National Patient Registry

Most children younger than 2 years who are hospitalized with symptoms of a respiratory infection will have a sample of nasal secretion taken (a nasopharyngeal aspirate) to confirm the diagnosis. Thirteen microbiological departments and the Statens Serum Institut perform and register microbiological identification of RSV infections in Denmark. Virus detection is the 'gold standard' for RSV diagnosis. There are reasons to believe that this may lead to under-diagnosis of RSV infection, however, because virus is only detectable during the first few days of infection. In Guinea-Bissau, for example, using an enzyme-linked immunosorbent assay to detect secretory RSV IgM, 38% of lower respiratory infections were associated with RSV, as opposed to the 17% identified using only an enzyme-linked immunosorbent assay for the antigen [45].

Every patient discharged from a Danish hospital is registered in the DNPR. Among the information entered into the DNPR are diagnoses, which have to be selected from the 10th revision of the International Statistical Classification of Diseases and Related Health Problems [46]. That classification has four RSV-specific diagnoses, as well as a rich variety of diagnoses that may cover the symptoms of an RSV infection. As noted by the Danish National Health Service, there is considerable variation in how different paediatric departments enter their diagnoses [47]. It is therefore important to know not only how RSV-positive cases are registered in DNPR but also how clinically suspected RSV cases are classified. To be able to use the DNPR for epidemiological studies, we need to identify the pattern of classification of diagnoses.

### The Danish Respiratory Syncytial Virus Database

**Figure 1:**
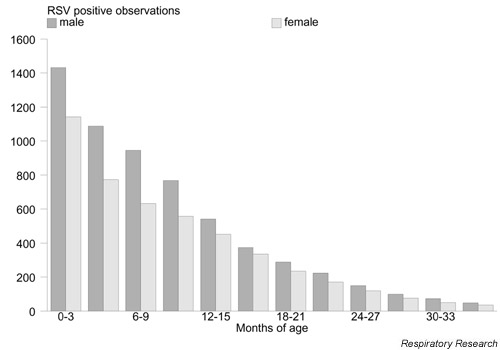
Distribution of sex and age in respiratory syncytial virus (RSV)-positive Danish children younger than 3 years.

Through the 13 microbiological departments and the Statens Serum Institut, we have established a database with information on RSV analyses and the personal identification numbers of people tested for RSV in Denmark since 1996. To date, the database contains information on 10,870 RSV-positive and 23,064 RSV-negative samples. Of the RSV-positive findings, 67% (7265) were in infants. The male:female ratio in RSV-positive findings is 1.3:1 (6117/4643; 110 missing values). Distributions of sex and age in the 10,458 RSV-positive findings in children younger than 3 years old are presented in Fig. 1.

Through the DNPR, we will collect hospitalization information on these individuals as well as on all those with RSV-specific diagnoses since 1996 who are not already in the laboratory register. The proposed analyses will determine the reliability of the RSV-specific categories in the DNPR and will indicate how well these cover the full range of RSV infections that lead to hospitalization. Furthermore, the study will provide a good basis for standardizing classification of RSV infections in the future. Above all, we intend to examine whether there has been an increase in RSV-hospitalized cases during the study period (1996–2001), as has been observed in the USA [6].

### Birth cohorts

The role of various biological, social and environmental factors will be studied through various population-based registers, the two major ones being the Danish National Birth Cohort (which has documented a large number of exposure variables) and the Danish child population in the CPR register. The main outcome to be evaluated will be hospitalization associated with RSV infection, as defined either by a positive RSV sample in the Danish RSV Database or by a reliable clinical diagnosis in the DNPR, as defined by the validation study described above. Nosocomial infection will be defined as RSV infection diagnosed after 4 or more days of an inpatient stay when hospitalization is for other reasons.

### The Danish National Birth Cohort

In 1997, the Danish Epidemiology Science Centre, Statens Serum Institut, initiated The Danish National Birth Cohort: 'Better health for mothers and children'. Since 1997, women have been invited to participate in the study if they speak Danish and intend to carry their pregnancies to term. At April 2002, approximately 95,000 pregnant women had been enrolled. Participation involves four computer-assisted telephone interviews in weeks 12 and 30 of pregnancy (1st and 2nd interviews), and 6 and 18 months after delivery (3rd and 4th interviews). Information on health, lifestyle, socioeconomic status throughout pregnancy, and delivery is collected. Data regarding feeding habits, diseases, medication, vaccinations, motor and cognitive development, anthropometrics, and day-care attendance are obtained on the child. The age at which illness is contracted is registered. Blood samples are collected from the pregnant women in weeks 12 and 24, and cord blood samples are obtained at birth.

### The Danish child population

Through the CPR, we will construct a cohort of children born in Denmark between 1996 and 2002 that can be linked with the laboratory database, as well as with the DNPR, in order to identify individuals who had severe RSV infection leading to hospitalization. It is estimated that this cohort will include around 390,000 children. Data regarding potential risk factors, protective factors and confounders will be obtained from the Medical Birth Registry, the DNPR and other public registers, including information on social conditions, day-care attendance, immunizations and medication.

Depending on the research question, the RSV hospitalization cases will be compared with controls selected within the same population groups on a case-control or a cohort basis to produce estimates of age-adjusted and sex-adjusted relative risks (odds ratio and relative risk) for hospitalization associated with different factors of interest. The choice between the Danish National Birth Cohort and this child population for the analyses will depend on different factors including quality of information on exposure, facility of access, and the prevalence of the factor under study.

### Sample size considerations

Based on two recent Danish studies that found the incidence of RSV infection requiring hospitalization to be 34/1000 for infants younger than 6 months and 14/1000 for those younger that 3 years, we estimate that 2% of children below 2 years of age will be hospitalized for RSV disease. With an annual birth cohort of 65,000 and 390,000 children born between 1996 and 2002, we are likely to identify approximately 7800 RSV-related hospitalizations during this period. This sample size should aid in the detection of even small additional risks in studies of the total population. Sample size for the studies involving biological materials will be calculated on the basis of pilot studies to determine the level of atopy and RSV antibodies in the current Danish population of mothers.

### Timetable

The study was initiated in January 2001, at which time we began data collection and the validation study, and will last until December 2005.

### Ethics

The study is noninterventional and does not require further contact with individuals once they are registered either at the microbiological departments or in the Danish National Birth Cohort. Therefore, further informed consent is not required. Only anonymous data will be reported.

## Abbreviations

CPR = Central Person Register; DNPR = Danish National Patient Registry; RSV = respiratory syncytial virus; Th = T-helper.

## References

[B1] Chanock R, Roizman B, Myers R (1957). Recovery from infants with respiratory illness of a virus related to chimpanzee coryza agent (CCA).. Am J Hyg.

[B2] Collins PL, Chanock RM, Murphy BR, Knipe DM, Howley PM (2001). Respiratory syncytial virus.. In Fields Virology.

[B3] Selwyn BJ (1990). The epidemiology of acute respiratory tract infection in young children: comparison of findings from several developing countries. Coordinated Data Group of BOSTID Researchers.. Rev Infect Dis.

[B4] Glezen P, Denny FW (1973). Epidemiology of acute lower respiratory disease in children.. N Engl J Med.

[B5] Meissner HC (1994). Economic impact of viral respiratory disease in children.. J Pediatr.

[B6] Shay DK, Holman RC, Newman RD, Liu LL, Stout JW, Anderson LJ (1999). Bronchiolitis-associated hospitalizations among US children, 1980–1996.. JAMA.

[B7] Kristensen K, Dahm T, Frederiksen PS, Ibsen J, Iyore E, Jensen AM, Kjaer BB, Olofsson K, Pedersen P, Poulsen S (1998). Epidemiology of respiratory syncytial virus infection requiring hospitalization in East Denmark.. Pediatr Infect Dis J.

[B8] Dessau RB, Schonheyder HC (1994). Respiratory syncytial virus infection. A frequent child disease in Denmark with annual outbreaks [in Danish].. Ugeskr Laeger.

[B9] de Sierra TM, Kumar ML, Wasser TE, Murphy BR, Subbarao EK (1993). Respiratory syncytial virus-specific immunoglobulins in preterm infants.. J Pediatr.

[B10] Fixler DE (1996). Respiratory syncytial virus infection in children with congenital heart disease: a review.. Pediatr Cardiol.

[B11] Abman SH, Ogle JW, Butler-Simon N, Rumack CM, Accurso FJ (1988). Role of respiratory syncytial virus in early hospitalizations for respiratory distress of young infants with cystic fibrosis.. J Pediatr.

[B12] Arnold SR, Wang EE, Law BJ, Boucher FD, Stephens D, Robinson JL, Dobson S, Langley JM, McDonald J, MacDonald NE, Mitchell I (1999). Variable morbidity of respiratory syncytial virus infection in patients with underlying lung disease: a review of the PICNIC RSV database. Pediatric Investigators Collaborative Network on Infections in Canada.. Pediatr Infect Dis J.

[B13] Hall CB, Powell KR, MacDonald NE, Gala CL, Menegus ME, Suffin SC, Cohen HJ (1986). Respiratory syncytial viral infection in children with compromised immune function.. N Engl J Med.

[B14] Falsey AR, Treanor JJ, Betts RF, Walsh EE (1992). Viral respiratory infections in the institutionalized elderly: clinical and epidemiologic findings.. J Am Geriatr Soc.

[B15] Falsey AR, Cunningham CK, Barker WH, Kouides RW, Yuen JB, Menegus M, Weiner LB, Bonville CA, Betts RF (1995). Respiratory syncytial virus and influenza A infections in the hospitalized elderly.. J Infect Dis.

[B16] Nachman SA, Navaie-Waliser M, Qureshi MZ (1997). Rehospitalization with respiratory syncytial virus after neonatal intensive care unit discharge: a 3-year follow-up.. Pediatrics.

[B17] Glezen WP, Paredes A, Allison JE, Taber LH, Frank AL (1981). Risk of respiratory syncytial virus infection for infants from low-income families in relationship to age, sex, ethnic group, and maternal antibody level.. J Pediatr.

[B18] Anderson LJ, Parker RA, Strikas RA, Farrar JA, Gangarosa EJ, Keyserling HL, Sikes RK (1988). Day-care center attendance and hospitalization for lower respiratory tract illness.. Pediatrics.

[B19] Trefny P, Stricker T, Baerlocher C, Sennhauser FH (2000). Family history of atopy and clinical course of RSV infection in ambulatory and hospitalized infants.. Pediatr Pulmonol.

[B20] Banzhoff A, Dulleck A, Petzoldt S, Rieger CH (1994). Salivary anti-RSV IgA antibodies and respiratory infections during the first year of life in atopic and non-atopic infants.. Pediatr Allergy Immunol.

[B21] Holberg CJ, Wright AL, Martinez FD, Ray CG, Taussig LM, Lebowitz MD (1991). Risk factors for respiratory syncytial virus-associated lower respiratory illnesses in the first year of life.. Am J Epidemiol.

[B22] Englund JA (1994). Passive protection against respiratory syncytial virus disease in infants: the role of maternal antibody.. Pediatr Infect Dis J.

[B23] Downham MA, Scott R, Sims DG, Webb JK, Gardner PS (1976). Breast-feeding protects against respiratory syncytial virus infections.. BMJ.

[B24] Eriksson M, Bennet R, Nilsson A (2000). Wheezing following lower respiratory tract infections with respiratory syncytial virus and influenza A in infancy.. Pediatr Allergy Immunol.

[B25] Castro-Rodriguez JA, Holberg CJ, Wright AL, Halonen M, Taussig LM, Morgan WJ, Martinez FD (1999). Association of radiologically ascertained pneumonia before age 3 yr with asthmalike symptoms and pulmonary function during childhood: a prospective study.. Am J Respir Crit Care Med.

[B26] McBride JT (1999). Pulmonary function changes in children after respiratory syncytial virus infection in infancy.. J Pediatr.

[B27] Sigurs N, Bjarnason R, Sigurbergsson F, Kjellman B, Bjorksten B (1995). Asthma and immunoglobulin E antibodies after respiratory syncytial virus bronchiolitis: a prospective cohort study with matched controls.. Pediatrics.

[B28] Bendelja K, Gagro A, Bace A, Lokar-Kolbas R, Krsulovic-Hresic V, Drazenovic V, Mlinaric-Galinovic G, Rabatic S (2000). Predominant type-2 response in infants with respiratory syncytial virus (RSV) infection demonstrated by cytokine flow cytometry.. Clin Exp Immunol.

[B29] Boelen A, Andeweg A, Kwakkel J, Lokhorst W, Bestebroer T, Dormans J, Kimman T (2000). Both immunisation with a formalin-inactivated respiratory syncytial virus (RSV) vaccine and a mock antigen vaccine induce severe lung pathology and a Th2 cytokine profile in RSV-challenged mice.. Vaccine.

[B30] Hanson LA, Dahlman-Hoglund A, Lundin S, Karlsson M, Dahlgren U, Ahlstedt S, Telemo E (1997). Early determinants of immunocompetence.. Nutr Rev.

[B31] Marci M, Vigano A, Trabattoni D, Villa ML, Salvaggio A, Clerici E, Clerici M (1996). Characterization of type 1 and type 2 cytokine production profile in physiologic and pathologic human pregnancy.. Clin Exp Immunol.

[B32] Prescott SL, Macaubas C, Smallacombe T, Holt BJ, Sly PD, Holt PG (1999). Development of allergen-specific T-cell memory in atopic and normal children.. Lancet.

[B33] van dV, Laan MP, Baert MR, de Waal MR, Neijens HJ, Savelkoul HF (2001). Selective development of a strong Th2 cytokine profile in high-risk children who develop atopy: risk factors and regulatory role of IFN-gamma, IL-4 and IL-10.. Clin Exp Allergy.

[B34] Marchant A, Goetghebuer T, Ota MO, Wolfe I, Ceesay SJ, De Groote D, Corrah T, Bennett S, Wheeler J, Huygen K, Aaby P, McAdam KP, Newport MJ (1999). Newborns develop a Th1-type immune response to *Mycobacterium bovis* bacillus Calmette-Guerin vaccination.. J Immunol.

[B35] Aaby P, Shaheen SO, Heyes CB, Goudiaby A, Hall AJ, Shiell AW, Jensen H, Marchant A (2000). Early BCG vaccination and reduction in atopy in Guinea-Bissau.. Clin Exp Allergy.

[B36] Benn CS, Jeppesen DL, Hasselbalch H, Olesen AB, Nielsen J, Bjorksten B, Lisse I, Aaby P (2001). Thymus size and head circumference at birth and the development of allergic diseases.. Clin Exp Allergy.

[B37] Droste JH, Wieringa MH, Weyler JJ, Nelen VJ, Vermeire PA, Van Bever HP (2000). Does the use of antibiotics in early childhood increase the risk of asthma and allergic disease?. Clin Exp Allergy.

[B38] Aaby P (1988). Malnutrition and overcrowding/intensive exposure in severe measles infection: review of community studies.. Rev Infect Dis.

[B39] Aaby P (1992). Influence of cross-sex transmission on measles mortality in rural Senegal.. Lancet.

[B40] Nielsen NM, Wohlfart J, Melbye M, Mølbak K, Aaby P (2002). Does cross-sex transmission increase the severity of polio infection? A study of multiple family cases.. Scand J Infect Dis.

[B41] The IMpact-RSV Study Group (1998). Palivizumab, a humanized respiratory syncytial virus monoclonal antibody, reduces hospitalization from respiratory syncytial virus infection in high-risk infants.. Pediatrics.

[B42] Meissner HC, Welliver RC, Chartrand SA, Law BJ, Weisman LE, Dorkin HL, Rodriguez WJ (1999). Immunoprophylaxis with palivizumab, a humanized respiratory syncytial virus monoclonal antibody, for prevention of respiratory syncytial virus infection in high risk infants: a consensus opinion.. Pediatr Infect Dis J.

[B43] American Academy of Pediatrics Committee on Infectious Diseases and Committee of Fetus and Newborn (1998). Prevention of respiratory syncytial virus infections: indications for the use of palivizumab and update on the use of RSV-IGIV.. Pediatrics.

[B44] Piedimonte G, King KA, Holmgren NL, Bertrand PJ, Rodriguez MM, Hirsch RL (2000). A humanized monoclonal antibody against respiratory syncytial virus (palivizumab) inhibits RSV-induced neurogenic-mediated inflammation in rat airways.. Pediatr Res.

[B45] Stensballe LG, Kofoed PE, Nante EJ, Sambo M, Jensen IP, Aaby P (2000). Duration of secretory IgM and IgA antibodies to respiratory syncytial virus in a community study in Guinea-Bissau.. Acta Paediatr.

[B46] World Health Organization (1992). International Statistical Classification of Diseases and Related Health Problems, 10th revision (ICD-10) Geneva: World Health Organization;.

[B47] (1999). Department of Medical Informatics, The Danish National Health Service. Guide to Speciality-Specific Coding and Registration of Clinical Diagnoses, 1st ed [in Danish] Copenhagen: The Danish National Health Service;.

